# Two decades of population genomics: will we ever agree on bacterial species?

**DOI:** 10.1186/s12915-023-01797-7

**Published:** 2024-01-26

**Authors:** William P. Hanage

**Affiliations:** Center for Communicable Disease Dynamics, Harvard T.H. Chan School of Public Health, Boston, MA USA

## Abstract

**We have never known more about the genetic variation that characterizes life on earth, which is stored in ever-growing databases, many of which are publicly accessible. Yet, an accessible database does not mean that information is readily usable or interpretable. Here, I consider how the last two decades of gene and genome sequencing have advanced our understanding specifically of pathogen variation and how the field might be revolutionized all over again — provided we are able to solve the challenges that have become evident as with the size of our databases.**

## A genomics revolution and the tip of the data iceberg

Since *BMC Biology* went live a couple of decades ago, a succession of technical advances have made genome sequencing ever cheaper and more accessible. And yet it is still worth pausing to appreciate that however enormous the advances have been, so much remains to be determined.

Twenty years ago, Sanger sequencing was the norm and the gold standard. I personally sequenced tens of thousands of loci from bacterial pathogens and would find electropherograms before my eyes when I shut them at night. More than once, I was told I had been muttering “G… A… G… C…” in my slumber. We stored those sequences in online databases that we thought were large at the time, having little with which to compare them.

It became clear that collecting lots of genetic data from many members of a population allowed us to ask new questions, and one was to examine the genetic variation associated with those awkward things we call “species.” The Bacteria are a Superkingdom of life in which the meaning of that word, never as secure as people might think [1], becomes truly unsteady. In 2005, we published an analysis of recombining bacteria we termed “fuzzy” species as a result of the way that isolates frequently contained divergent sequence that was characteristic of other bacteria in closely related but distinct “species” [2].

This paper involved phylogenies containing some hundreds of tips, constructed from alignments a few thousand base pairs in length, with one of the then emerging Bayesian methods that promised the ability to handle extremely complex questions and datasets. Nevertheless, it took well over a week to run. And more than once, I would find the chains had *still* not converged and curse the method before realizing the problem lay instead with my input file, which the program was reasonably treating as advertised.

At that time, genomics was about to be revolutionized by the introduction of technologies collectively termed “next generation” [3]. While this coining leaves much to be desired, in that just as all art has been contemporary at some point, so has all sequencing technology been next generation when it was first developed, the impact was still profound. We were less likely to be talking about sequencing genes than sequencing genomes. I recall a meeting discussing the mushrooming data storage requirements at which one attendee suggested that rather than storing all the genomes that were sequenced as bytes, it would be more economical to invest in freezers to store the DNA and sequence it anew if you needed it. I’m still not sure they were joking.

Since then, online databases of pathogen genetic data have grown, developing their own gravitational pull as researchers worldwide are eager to submit their data to them and compare with the work of their colleagues. Recently, they have found their way into the public consciousness and even news headlines [4]. As I type, the GISAID platform contains sequence and data from more than 15… hang on, actually make that 16 *million* SARS-CoV-2 genomes! Even more astonishing, thanks to techniques like MASH [5], we have ways to represent their relatedness that will not bring processors to their metaphorical knees.

And yet those headlines indicate issues of ethics and governance that have accompanied the inexorable rise of the databases. Alongside which are the knotty questions of which genomes exactly get sequenced? We have good samples from some parts of the world — the rich parts. Others are woefully understudied even when we know they are a focus of morbidity and mortality. And it is not only the bacteria that matter. Our knowledge of the genomic variation of all the viruses that could infect us is not only the tip of the iceberg. It may be better described as the thin layer of evaporating water molecules escaping the sheen of ice melt atop the iceberg as it thaws a little in the weak noonday sun.

The issues do not stop there. Most of the time when we speak of “whole genome sequence,” we mean a draft genome. In the great majority of cases, we do not have genomes that start with an origin of replication and continue uninterrupted all the way to their end (or around a circular chromosome). Instead, we have “contigs,” meaning a chunk of contiguous sequence, of varying size — the fewer of them and the bigger they are the better because it implies more of the genome has been captured. In between them are regions that cannot be figured out for whatever reason — low coverage, poor quality, and so on. Even within contigs, we can find regions where the exact sequence cannot be determined, and bases are represented as Ns. For things where we rely on amplicon sequencing like many viruses, uncertainty creeps in as the genomes evolve and primers bind less well. Imperfect sequencing forces decisions that may not always be reflected in the genomes that find their way into databases. The base at any position where the bioinformatics have not delivered a clear and unambiguous signal could be called as that of the reference genome, or an ancestral sequence (which will systematically obscure changes in difficult regions to sequence), or as an N, or simply be “masked” — part of a process for filtering out complicated regions of the genome. Differences in the exact pipeline and criteria for variant calling and genome assembly can readily confuse other users and indeed have done [6].

There is also a growing appreciation that a single consensus sequence cannot adequately capture the variation present in a population. In some cases, this has proved a new and useful source of data. My lab has used it to infer when infections are linked by transmission [7]. The phenomenon of heteroresistance is another example; a population descended from a single clone can rapidly generate variable susceptibility to an antibiotic by changing the copy number of the resistance loci [8].

In other cases, consistently variable positions in the genome may reflect some form of balancing selection in the environment from which the sample was collected. Such phase variation is known in some bacteria but may be more common than suspected. In this case, it is sobering to note how much of microbiology has involved culturing organisms to get enough genetic material to sequence, and culture will naturally remove any such variation that is not selected by the media! Genomes assembled from metagenomic data may address this problem in time, as will the widespread use of long-read and single-cell sequencing technologies. Yet, these will come with their own challenges when it comes to data collection, analysis, and storage.

In short, 20 years ago, using sequence data to delineate species seemed an obvious thing to do. The use of genomes ought to have made that simpler, except it often has not. Now, we have more sequence data than could easily have been imagined; we still argue about species [9] and still face many of the same problems in terms of collecting adequate metadata and database management. In some cases, we have allowed our technological ability to *do* to outpace our ability to think about what is worth doing.

And plasmids, oh my goodness. We've not mentioned plasmids! In bacteria, these are some of the most interesting parts of the genome, and given that they are not part of the main chromosome, they have been very technically demanding to sequence. Plasmids are highly diverse, highly mobile both within and between host “species,” and demand attention due to their importance in spreading genes involved in virulence and drug resistance. When it comes to plasmids, we are only getting started.

## Data Availability

Not applicable.
